# Supplementary material for: Single-cell transcriptome analysis reveals TOX as a promoting factor for T cell exhaustion and a predictor for anti-PD-1 responses in human cancer
Source: Genome Med. 2020 Feb 28;12:22. doi: 10.1186/s13073-020-00722-9 (PMC7048139; doi:10.1186/s13073-020-00722-9)
Supplement: Supplementary file 2 — Additional file 2:Table S2. The baseline characteristics of clinical samples subjected to flow cytometric analysis. Table S3. The baseline clinicopathological characteristics of patients receiving anti-PD-1 therapy. Table S4. Clinical outcomes of patients with non-small cell lung cancer (NSCLC) receiving anti-PD-1 therapy at the Yonsei Cancer Center. Figure S1. Correlation between protein expression levels of transcription factors (TFs) and immune checkpoint (IC) molecules in the tumor-infiltrating (TI) CD8+ T cells in human non-small cell lung cancer (NSCLC). Figure S2. Distribution of cells from each patient between PDCD1-high and PDCD1-low subsets. Figure S3. Correlation between TOX expression and severity of tumor-infiltrating (TI) CD8+ T-cell exhaustion in various mouse tumors. Figure S4. Correlation between protein expression levels of transcription factors (TFs) and immune checkpoint (IC) molecules in the tumor-infiltrating (TI) CD8+ T-cells in various mouse tumors. Figure S5. Overall survival analysis of The Cancer Genome Atlas (TCGA) cohort of patients with bladder cancer, head-and-neck cancer, sarcoma, and uterine cancer exhibiting varying TOX expression levels in the tumor-infiltrating T cells. Figure S6. Relative abundance of T cells in patients receiving anti-PD-1 therapy. Figure S7. TOX-dependent regulation of the expression of NR4A1 in the tumor-infiltrating (TI) CD8+ T cells in human non-small cell lung cancer (NSCLC). [file 13073_2020_722_MOESM2_ESM.pdf]

Single-cell transcriptome analysis reveals TOX as a promoting factor for T-cell exhaustion and a predictor for anti-PD-1 responses in human cancer

Kyungsoo Kim, Seyeon Park, Seong Yong Park, Gamin Kim, Su Myeong Park, Jae-Won Cho, Da Hee Kim, Young Min Park, Yoon Woo Koh, Hye Ryun Kim, Sang-Jun Ha, and Insuk Lee

Contents

Table S2. The baseline characteristics of clinical samples subjected to flow cytometric analysis.

Table S3. The baseline clinicopathological characteristics of patients receiving anti-PD-1 therapy.

Table S4. Clinical outcomes of patients with non-small cell lung cancer (NSCLC) receiving anti-PD-1 therapy at the Yonsei Cancer Center.

Fig S1. Correlation between protein expression levels of transcription factors (TFs) and immune checkpoint (IC) molecules in the tumor-infiltrating (TI) $CD8^+$ T cells in human non-small cell lung cancer (NSCLC).

Fig S2. Distribution of cells from each patient between *PDCD1*-high and *PDCD1*-low subsets.

Fig S3. Correlation between TOX expression and severity of tumor-infiltrating (TI) $CD8^+$ T-cell exhaustion in various mouse tumors.

Fig S4. Correlation between protein expression levels of transcription factors (TFs) and immune checkpoint (IC) molecules in the tumor-infiltrating (TI) $CD8^+$ T-cells in various mouse tumors.

Fig S5. Overall survival analysis of The Cancer Genome Atlas (TCGA) cohort of patients with bladder cancer, head-and-neck cancer, sarcoma, and uterine cancer exhibiting varying TOX expression levels in the tumor-infiltrating T cells.

Fig S6. Relative abundance of T cells in patients receiving anti-PD-1 therapy.

Fig S7. TOX-dependent regulation of the expression of NR4A1 in the tumor-infiltrating (TI) $CD8^+$ T cells in human non-small cell lung cancer (NSCLC).

Table S2. The baseline characteristics of clinical samples subjected to flow cytometric analysis.

Characteristics	Cancer type	
	Head-and-neck cancer	Non-small cell lung cancer
Number of patients	15	35
Age in years (median, range)	60.4 (37−76)	60.8 (44−75)
Sex		
Male	12 (80.0%)	24 (68.6%)
Female	3 (20.0%)	11 (31.4%)
Primary site		
Oropharyngeal cancer	7 (46.7%)	NA
Hypopharyngeal cancer	2 (13.3%)	NA
Oral cavity cancer	6 (40.0%)	NA
Lung cancer	NA	35 (100%)
Histology		
Adenocarcinoma	0 (0%)	27 (77.1%)
Squamous cell carcinoma	15 (100%)	8 (22.9%)
Mutational status		
EGFR mutation	NA	15 (42.9%)
Human papillomavirus (HPV) status		
Positive	6 (40.0%)	NA
Negative	9 (60.0%)	NA
Stage		
I	1 (6.7%)	17 (48.6%)
II	5 (33.3%)	13 (37.1%)
III	3 (20.0%)	5 (14.3%)
IV	6 (40.0%)	0 (0%)

NA, not available

Table S3. The baseline clinicopathological characteristics of patients receiving anti-PD-1 therapy.

	Non-small cell lung cancer (N = 16)
Median age in years (range)	64 (34–76)
Sex	
Male	13 (81.2%)
Female	3 (18.8%)
Smoking history	
Never smoker	2 (12.5%)
Current/former smoker	14 (87.5%)
ECOG performance status	
≤1	14 (87.5%)
>1	2 (12.5%)
Histology	
Adenocarcinoma	8 (50%)
Squamous cell carcinoma	8 (50%)
PD-L1 positivity	
Positive	4(25.0%)
Negative	10 (75.0%)
N/A	2 (12.5%)
Types of drug	
Nivolumab	16 (100%)
Responsiveness to anti-PD-1 therapy	
Response	5 (31.2%)
Non-response	11 (68.8%)

ECOG, Eastern Cooperative Oncology Group; N/A, not available;

Table S4. Clinical outcomes of patients with non-small cell lung cancer (NSCLC) receiving anti-PD-1 therapy at the Yonsei Cancer Center.

Patient ID	Responder (R) or non-responder (NR)
Dis_01	NR
Dis_02	R
Dis_03	NR
Dis_04	R
Dis_05	NR
Dis_06	NR
Dis_07	NR
Dis_08	NR
Dis_09	NR
Dis_10	R
Dis_11	NR
Dis_12	NR
Dis_15	R
Dis_16	NR
Dis_17	R
Dis_18	NR

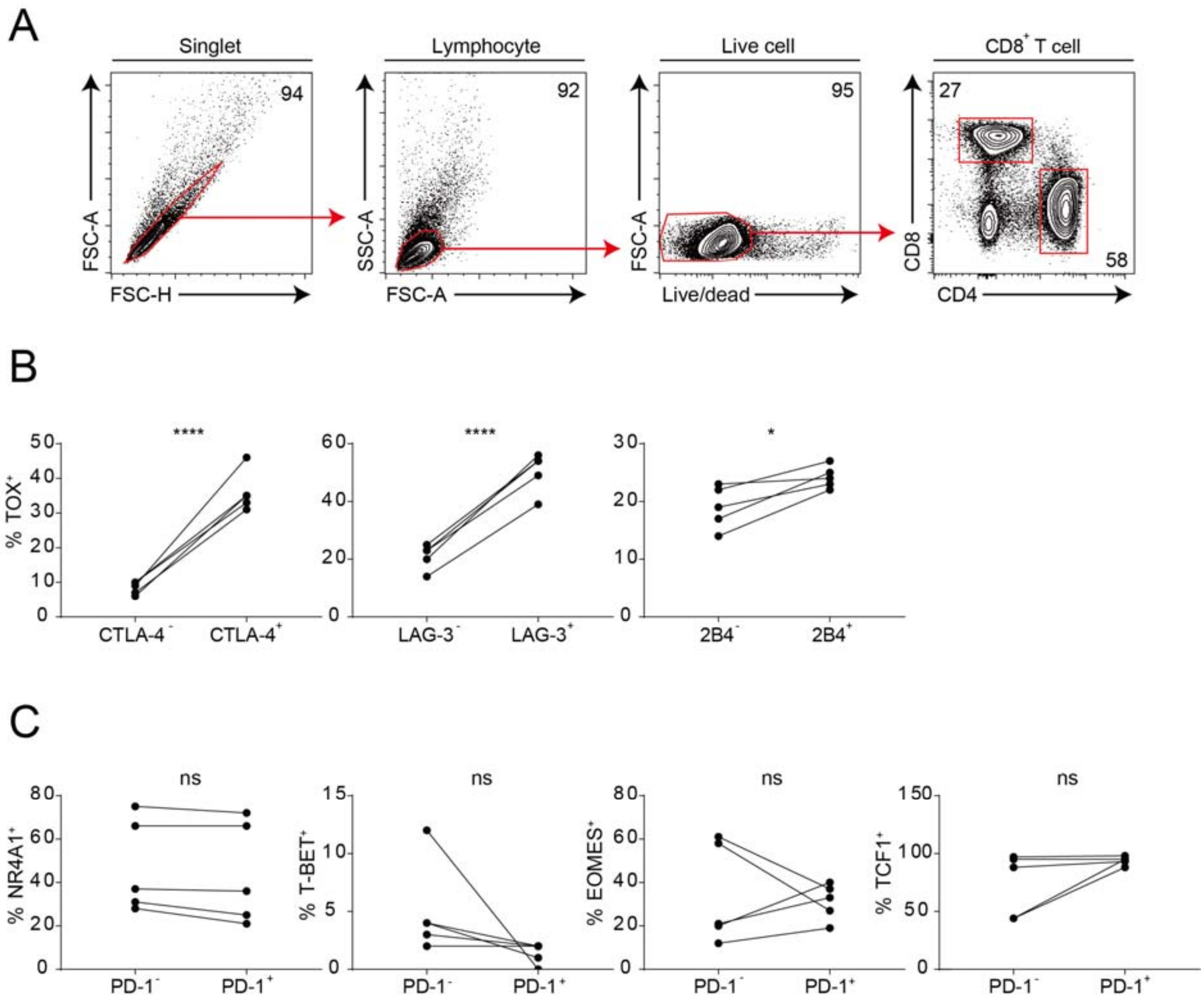

Fig S1. Correlation between protein expression levels of transcription factors (TFs) and immune checkpoint (IC) molecules in the tumor-infiltrating (TI) CD8$^+$ T cells in human non-small cell lung cancer (NSCLC)

(A) Gating strategy to identify the TI CD8$^+$ T cells from human tumors. Singlets are delineated by forward scatter-height (FSC-H) against forward scatter-area (FSC-A). Gating the plot by forward scatter-area (FSC-A) versus side scatter-area (SSC-A) indicates lymphocytes. Dead cells are then excluded by forward side scatter-area (FSC-A) and LIVE/DEAD™ Stain Kit. After gating the live cells, the CD8$^+$ T-cell population is identified based on CD8 and CD4 expression. (B) Percentage of TOX$^+$ cells in the two subpopulations of TI CD8$^+$ T cells expressing or not expressing a specific IC molecule. (C) Percentage of TF-positive cells in the TI CD8$^+$ T cells with or without PD-1 expression. Each line in the graph of both (B) and (C) indicates data derived from the same tumor tissue of each individual patient. ns, not significant; $*P < 0.05$; $**P < 0.01$; $****P < 0.0001$. All statistical analyses were performed using the unpaired Student's *t*-test.

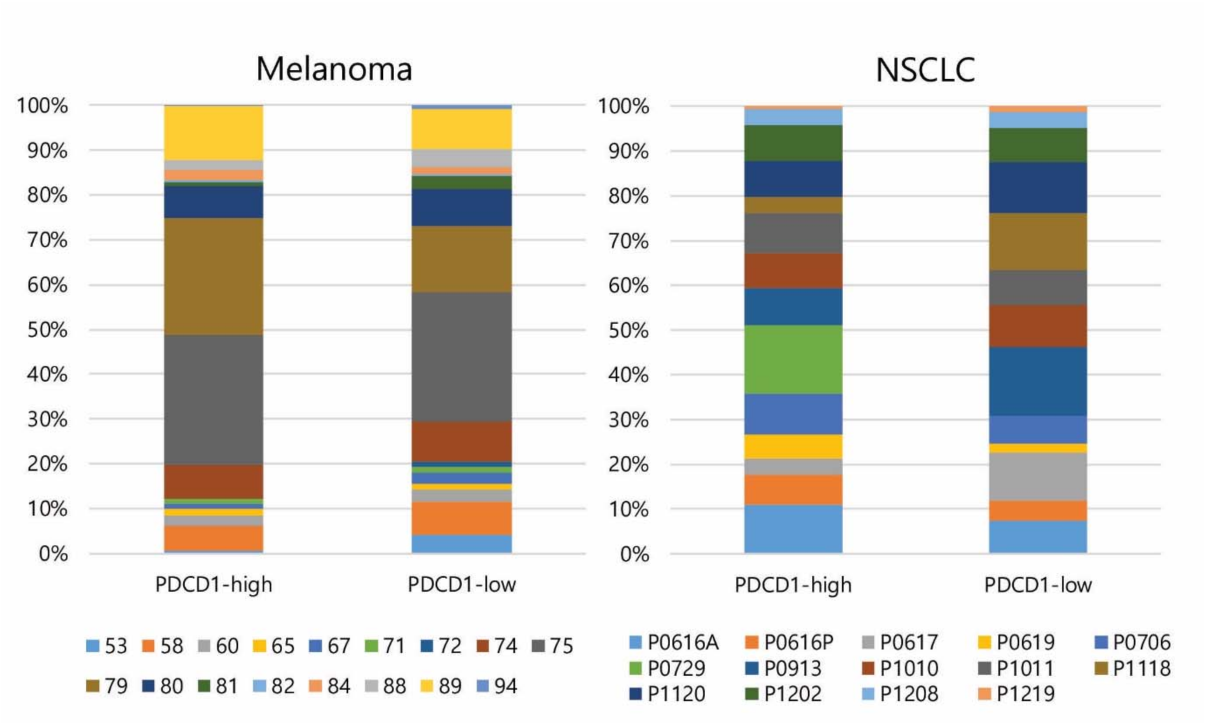

Fig S2. Distribution of cells from each patient between *PDCD1*-high and *PDCD1*-low subsets.

Proportion of cells from each patients in *PDCD1*-high and *PDCD1*-low was analyzed for the single-cell transcriptome datasets derived from melanoma and NSCLC.

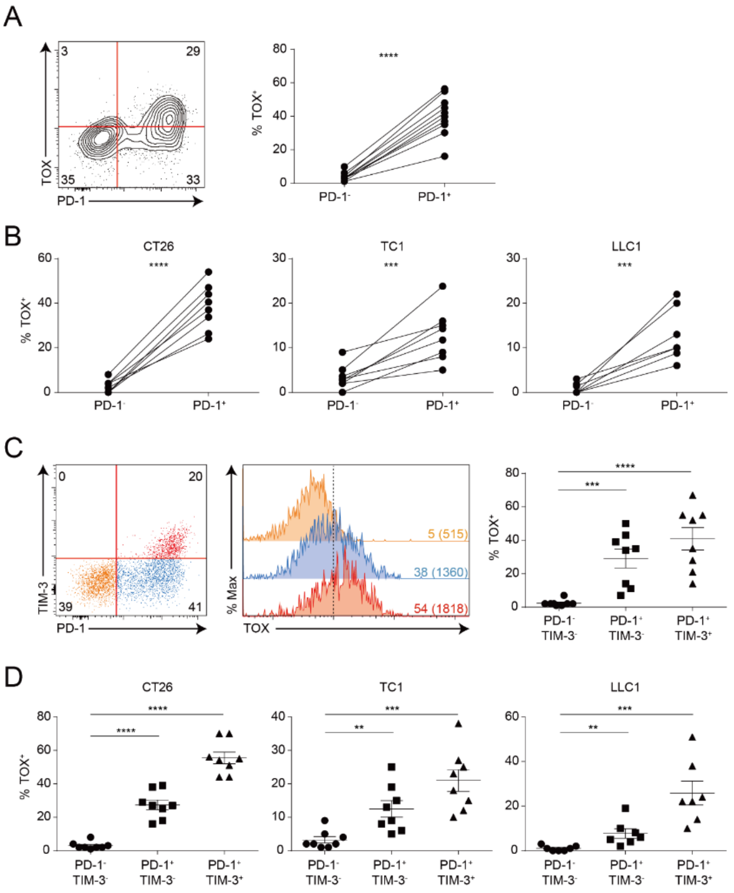

Fig S3. Correlation between TOX expression and tumor-infiltrating (TI) CD8+ T-cell exhaustion severity in various mouse tumors

(A-C) Flow cytometric analysis of the TI CD8+ T cells isolated from tumor tissues of mice bearing MC38, CT26, TC1, or LLC1 tumor. (A) Representative plot showing the co-expression of TOX and PD-1 in the TI CD8+ T cells from MC38 tumor (left) and percentage of TOX+ cells in the TI CD8+ T cells with or without PD-1 expression (right). (B) Percentage of TOX+ cells in TI CD8+ T cells with or without PD-1 expression from mouse tumors including CT26, TC1, and LLC1. Each line in the graph of both (A) and (B) indicates the data derived from the same tumor tissue of each individual mouse. (C) TOX protein levels in three subsets of the TI CD8+ T cells exhibiting varying exhaustion severity levels: PD-1-TIM-3- (orange), PD-1+TIM-3- (blue), and PD-1+TIM-3+ (red). TI CD8+ T cells were derived from MC38 tumor. Percentage of TOX-expressing cells in each subset is described in the histogram and mean fluorescence intensity (MFI) for TOX expression in each subset is indicated in parenthesis. A dashed line represents the boundary separating the expression of TOX protein. Distribution of TOX-expressing subsets of the TI CD8+ T cells across MC38 tumor samples is summarized in grouped scattered plots. (D) The distribution of TOX-expressing subsets of TI CD8+ T cells across tumor samples from CT26, TC1, and LLC1 is summarized in grouped scattered plots. Data shown in (A-D) are summarized from two independent experiments. n = 8-10 mice per group. ns, not significant; *$P < 0.05$; **$P < 0.01$; ***$P < 0.001$; ****$P < 0.0001$. All statistical analyses were performed using the unpaired Student's t-test.

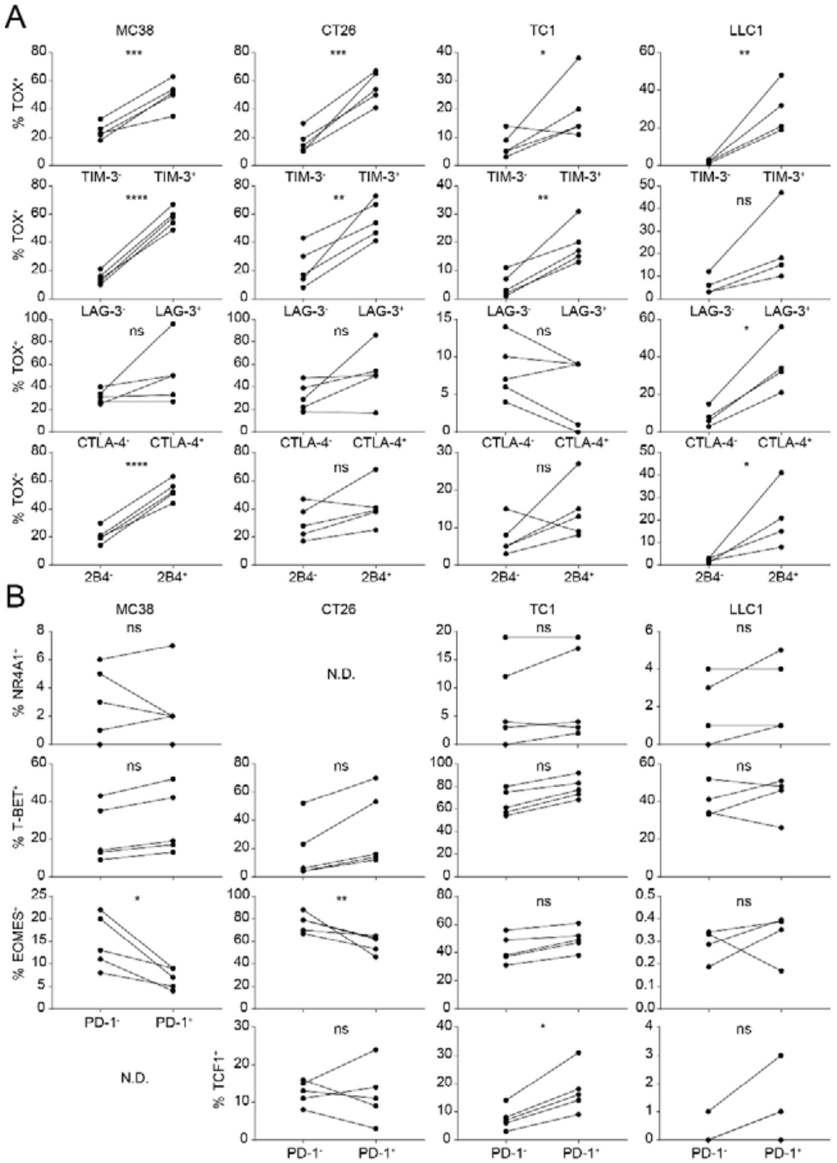

Fig S4. Correlation between protein expression levels of transcription factors (TFs) and immune checkpoint (IC) molecules in the tumor-infiltrating (TI) CD8^{+} T cells of various mouse tumors

(A) Percentage of TOX^{+} cells in the two subpopulations of the TI CD8^{+} T cells expressing or not expressing a specific IC molecule. Protein expression of IC molecules, such as TIM-3, LAG-3, CTLA-4, and 2B4 in the TI CD8^{+} T cells was measured by flow cytometry. (B) Percentage of cells expressing TFs, such as Nr4a1, T-bet, Eomes, and Tcf1 in the two subpopulations of TI CD8^{+} T cells with or without PD-1 expression. Mouse tumor tissues used in (A) and (B) were derived from MC38, CT26, TC1, or LLC1 tumor-bearing mice. Each line in the graph of both (A) and (B) indicates the data derived from the same tumor tissue of each individual mouse. n = 4-5 mice per group. ns, not significant; *P < 0.05; **P < 0.01; ***P < 0.001; ****P < 0.0001. All statistical analyses were performed using the unpaired Student's t-test.

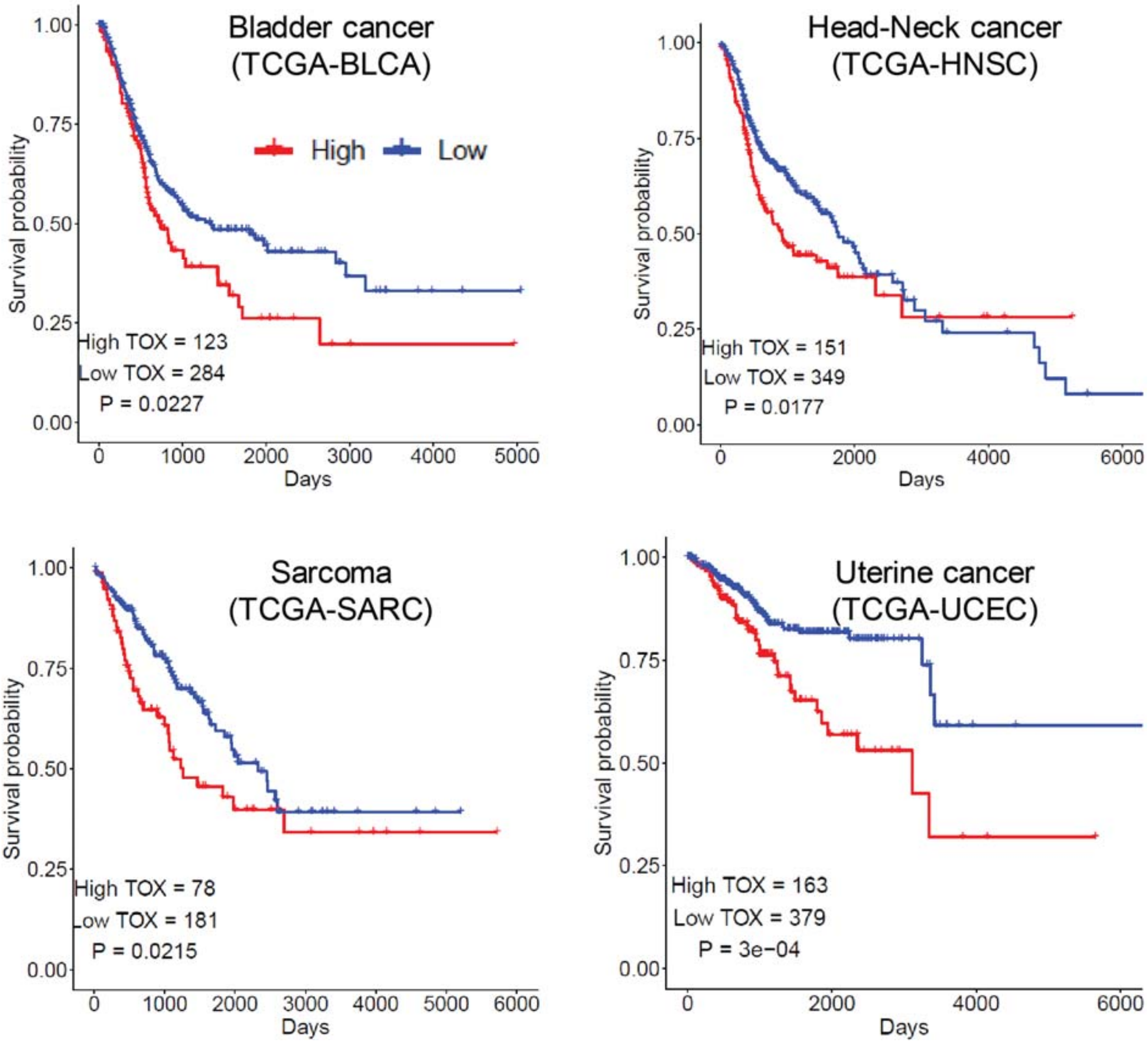

Fig S5. Overall survival analysis of The Cancer Genome Atlas (TCGA) cohorts of patients with bladder cancer, head-and-neck cancer, sarcoma, and uterine cancer exhibiting varying TOX expression levels in the tumor-infiltrating T cells.

Patients were classified into high-*TOX* for those exhibiting top 30% *TOX* expression level and low-*TOX* for the rest.

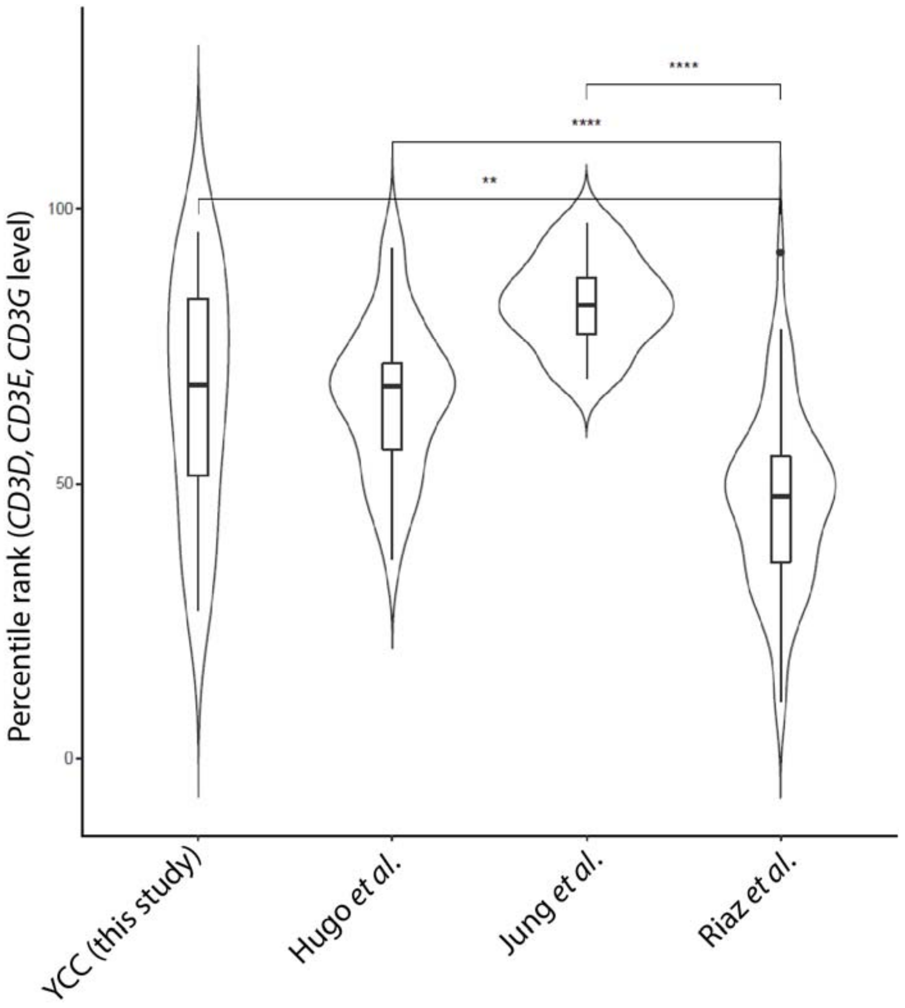

Fig S6. Relative abundance of T cells in patients receiving anti-PD-1 therapy. Geometric mean expression levels of CD3D, CD3E, and CD3G were used to estimate the relative abundance of T cells in the tumor. The percentile ranks of the mean expression levels of CD3D, CD3E and CD3G were compared among the three cohorts of patients receiving anti-PD-1 therapy. The estimated T cell abundance for the patients from Riaz et al. is markedly lower than that for the other cohorts.

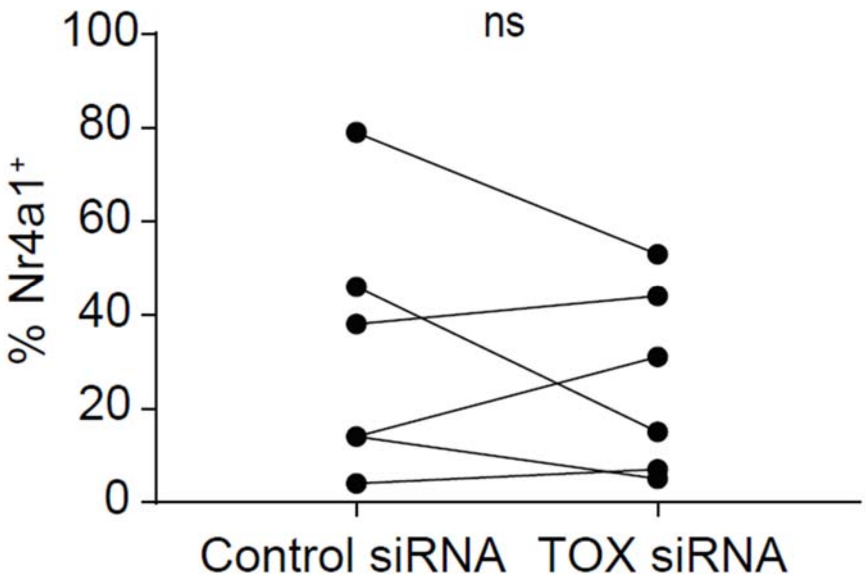

Fig S7. TOX-dependent regulation of the expression of NR4A1 in the tumor-infiltrating (TI) CD8$^+$ T cells of human non-small cell lung cancer (NSCLC). The expression level of NR4A1 in the TI CD8$^+$ T cells of human NSCLC after *TOX* mRNA knockdown. Each line in the graph indicates the data derived from the same tumor tissue of each individual patient. ns, not significant;
